# Narrative Identity within Mentalization-Based Group Therapy for Adolescents: A Feasibility Study

**DOI:** 10.3390/children10050854

**Published:** 2023-05-10

**Authors:** Majse Lind, Lennart Kiel, Sune Bo Hansen, Mie Sedoc Jørgensen, Erik Simonsen

**Affiliations:** 1 Department of Communication and Psychology, Aalborg University, Nordkraft, Teglgårds Plads 1, 9000 Aalborg, Denmark; 2 Department of Psychology, Aarhus University, 8000 Aarhus, Denmark; lennartkiel@psy.au.dk; 3 Department of Psychology, University of Copenhagen, 1353 Copenhagen, Denmark; sune.bo@rsyd.dk; 4 Psychiatric Research Unit, Mental Health Services of Region Zealand, Region Zealand, 4200 Slagelse, Denmark; 5 Psychiatry East, Mental Health Services of Region Zealand, 4000 Roskilde, Denmark; es@regionsjaelland.dk; 6 Department of Clinical Medicine, University of Copenhagen, 2200 Copenhagen, Denmark

**Keywords:** storying, narrative themes, mentalization-based group therapy, MGAB, adolescence, LPFS, agency, communion

## Abstract

Borderline personality disorder (BPD) is characterized by severe disturbances related to understanding oneself and other people and can be reliably detected and treated in adolescence. In this feasibility study, we aimed to focus on the features of, and changes in, narrative identity throughout the course of Mentalization-Based Treatment in Groups (MBT-G) for adolescents with BPD. Six female patients (*M* = 15.2, SD = 0.75) joined between 16 and 31 (*M* = 23.83) MBT g sessions. The narrated events within each session across sessions were coded for themes of agency and communion and the narrated reactions were coded for personality functioning. The patients and their parents also completed several self-report measures before and after therapy. Themes of diminished agency and communion were identified, with communion as the dominating theme. When comparing the patients’ first five sessions with their last five sessions, there was an increase in themes related to agency and decreased in communion. The narrated reactions were dominated by themes related to thwarted self-functioning and primarily identity, although intimacy was also present. Patients improved in terms of self-reported functioning and internalizing and externalizing behavior before and after end of treatment. The importance of narration in BPD (group) therapy is discussed alongside clinical implications.

## 1. Introduction

Borderline personality disorder (BPD) is a severe mental illness characterized by a persistent pattern of unstable emotion regulation, impulse control, self-image, and interpersonal challenges [1]. A compelling body of research justifies that a valid and reliable diagnosis of BPD can be made already in adolescence [2], possibly with an even higher prevalence than in adulthood [3]. On this ground, both researchers and clinicians have stressed the importance of early intervention in BPD to prevent severity over time [4]. Among adults with BPD, Mentalization-based therapy (MBT) has shown efficacy, especially in reducing psychiatric symptoms associated with BPD as well as related comorbidity (see [5] for a review). Among adolescents manifesting BPD, however, a paucity of evidence on the effect of treatment in general [6] and in MBT specifically [7] is the status quo. A recently published review and metanalysis [6] shows that psychotherapy for adolescents with BPD contributes to diminished BPD pathology and self-harm, with effects lingering over time when compared to control treatment. However, the review itself has been criticized for several methodological issues and results should be interpreted with some caution [8]. The uncertainty of treatment effectiveness and lack of evidence-based guidelines on psychotherapy to treat BPD motivated a Danish research group to first conduct a pilot study [9] following an RCT study [10] comparing mentalization-based group therapy with treatment as usual for adolescents with BPD. Although the pilot study displayed promising improvements on a number of psychological parameters (e.g., general psychopathology), the RCT failed to exhibit any significant improvements in the mentalization-based group compared to the control condition, and this trend remained steady across follow-ups [11]. However, these results are based on self-report and semi-structured clinical interviews, and codable features within the MBT sessions have not yet been analyzed.

With the dimensional shift in the personality disorder field, self-understanding and other-understanding have come to constitute the very heart of the disorder (e.g., [12]), with identity as one of the central building blocks [13]. Identity often takes the form of a story—a narrative identity—understood as the dynamic and evolving story the person tells about the past, present, and presumed future [14]. The overall life story, as well as smaller stories from the person’s life, contribute to a sense of self-continuity and stability as they provide content and interpretation connecting who one once was, who one currently is, and who one may become in the future through rich phenomenological lenses [14]. With the steadily dominating dimensional paradigm, narrative identity has never been more salient in the context of PD (see [15]). ICD-11 particularly highlights the importance of identity as “stable and coherent” [16], making the temporal aspect of identity, i.e., narrative identity, very important. With the preservation of a borderline pattern qualifier in ICD-11, research on BPD specifically will remain relevant with the dimensional shift. Lind and colleagues [15,17] have advocated for the importance of integrating narrative identity within the conceptualization and intervention of PDs as we enter the dimensional era. A compelling body of literature has already shown how narrative identity is disturbed within multiple domains in individuals suffering from personality disorders, with the vast majority of research focusing on adults manifesting BPD (see [18] for a review). Overall, adults with (B)PD construct narrative identities disturbed in the broader domains related to structure (e.g., less coherency), motivational/affective themes (e.g., negative emotional tone and diminished themes of agency and communion), and reasoning (negative meaning-making/causal connections). More recently, researchers have stressed the importance of studying narrative identity and BPD in adolescence [15,19], since this is the developmental period in which narrative identity emerges [20] either in adaptive or maladaptive ways. Indeed, research has shown that narrative identity begins to go awry already in adolescents manifesting BPD features [18]. Lind and colleagues displayed associations between elevated BPD features and less coherent narrative identities [21] and more diminished themes of communion (e.g., love, companionship, togetherness) and agency (e.g., mastery, achievement) in inpatient adolescents [22]. The studies indicate that adolescents with BPD have started to construct maladaptive narrative identities related to structure and motivational/affective themes. With the dimensional shift in PD, recent studies have examined, though failed to find, associations between the Levels of Personality Functioning Scale (LPFS, [23]) and aspects of narrative identity [24] in PD, while others have successfully rated [25,26] or coded [27] LPFS based on narrative identity transcripts. However, no study has yet coded LPFS based on narrative identity material in adolescents with BPD.

With the compelling literature linking disturbed narrative identity to BPD in adolescence, examining narrative identity more closely within and across therapeutic settings seems like an obvious next step. Although this has been highlighted by many [15,18], specifically in the context of ICD-11 [15,28], research in this area is so far almost non-existent. One study showed that adults with BPD increased significantly in narrative agency after one year of psychodynamic therapy (e.g., MBT), compared to a healthy control group [29]. That is, adults with BPD started crafting stories from their lives with an increased sense of authorship, ownership, independence, and mastery of life instead of feeling victimized by life circumstances, coincidences, and other people. Other aspects of narrative identity did not change significantly after therapy. Unfortunately, although adolescence is a critical developmental period for crafting narrative identity, and a time in which interventions are particularly important and advantageous [19], no study has yet examined narrative identity or LPFS related to the narrated events as they unfold within therapy in adolescents.

### The Present Study

The main aim of this feasibility study was to code significant events brought up in MBT g for adolescents with BPD. The events were coded for themes of agency and communion as they unfolded and developed throughout the therapy. The reactions to the events were further coded for LPFS themes (i.e., identity integration, self-direction, empathy, intimacy). No study has yet examined narrative identity themes and themes related to LPFS as they appear dynamically in BPD group therapy to adolescents, and therefore no previous hypotheses were posed. Both individual patient profiles and group tendencies were explored. Dimensional measures of BPD, externalizing and internalizing behavior, participant and parent reports of depression, and global functioning were also assessed before and after treatment.

## 2. Method

### Participants and Procedure

This study is part of a larger, M-GAB RCT with a total of 111 adolescents with BPD or subthreshold BPD (i.e., meeting at least four DSM-5 BPD criteria, [10]). The study was approved by the Regional Ethics Committee of Zealand (no: SJ-371) on 06/14-2011. Adolescents were assessed for a number of BPD criteria using the semi-structured Childhood Interview for DSM-IV Borderline Personality Disorder (CI-BPD, [30]). Readers are referred to the study protocol for details of the assessment instruments [9], and to the papers reporting outcomes at end of treatment [10], as well as the three- and twelve-month follow-ups [11]. Adolescents between 14 and 17 years at inclusion received MBT-G consisting of 37 weekly sessions (90 min.) in slow-open groups with a maximum of eight patients. The therapists who delivered the MBT were trained and supervised psychologists and psychiatrists. All the MBT-G sessions were videotaped and 10% were randomly selected for ratings for adherence to MBT-G, and showed “good-enough” adherence [10].

For the current feasibility study one therapy group was selected for an in-depth, first-person narrative analysis based on video recordings. The group consisted of seven female patients being enrolled continuously. One of the patients was included late in the group and was not a participant in the original study, for which reason she is not included in the current analyses. As such, this study investigates a total of six female patients ranging from 14 to 16 years of age (*M* = 15.2, SD = 0.75), who joined between 16 and 31 (*M* = 23.83, SD = 6.31) MBT-G therapy sessions.

## 3. Measures

### 3.1. Narrative Analysis and Coding of Video Recorded Therapy Sessions

For the narrative analyses, video recordings of group therapy sessions were coded by the second author after the first and second author had reached acceptable interrater reliability based on 20% of all the narrated events (Kappa = 0.71, *p* < 0.001) and the narrated reactions (Kappa = 0.75, *p* < 0.001). The coding focused specifically on the personal life event each adolescent was asked to identify and elaborate on within each session. For each patient, within each session, across sessions, the main theme for every event was first identified and thereafter the related reaction to the event was coded.

**Coding the main events: agency and communion.** The patients’ storied events were analyzed and coded based on McAdams’ [31] coding scheme of agency and communion themes for narrated autobiographical episodes. Agency-related events involve stories with a focus on achievement or responsibility, acquisition of new abilities, status, control, etc. Communion-related events comprise stories concerning friendship or love, receiving, or giving support, and in general establishing and maintaining relationships. The central thematic plot in each event was detected and the presence/absence of agency and communion was coded using a dichotomous format of 0 (absent) to 1 (present). A given event could only be scored as having either agency or communion as the primary plot, not both. Storied events with a thematic plot that was neither agency nor communion were coded as “other” (i.e., medical consultations, starting new medication, etc.). When adolescents were attending a session without sharing an event, it was coded as an “unidentified event”. To examine the proportion of absence/presence of agency, communion and unidentified events, scores were summed across all sessions. Each participant received a total score (ratio %) for each type of event based on each patient’s total number of sessions including sessions where a participant was present without narrating an event (i.e., unidentified events).

**Coding the reaction to the event: Personality functioning.** To examine the patients’ reactions to the narrated events, we used a newly developed coding system for self–other understanding inspired by the Level of Personality Functioning Scale (LPFS) in the Alternative Model of Personality Disorder [1,27]. The coding system covers four major narrative themes (Identity Integration, Self-Direction, Empathy, and Intimacy), with a total of twelve subthemes. Three subthemes are related to the major theme of Identity Integration: (1) experience of oneself as coherent, with clear boundaries between self and others, (2) sense of self-esteem, and (3) ability to regulate emotional experience. Three subthemes are related to the major theme of Self-Direction: (4) striving for meaningful, coherent short-term and life goals, (5) utilizing constructive and prosocial internal standards of behavior, and (6) the ability to self-reflect productively. Three subthemes related to the major theme of Empathy: (7) comprehension and appreciation of others’ experiences, (8) tolerance of others’ perspectives, and (9) understanding the effect of one’s own behavior on others. Lastly, three subthemes are related to the major theme of Intimacy: (10) the depth and/or duration of connection with others, (11) desire and capacity for closeness, and (12) reciprocity and mutuality of regard reflected in interpersonal behavior. The narrated reactions were categorized as belonging to one of the four main categories: Identity integration, Self-direction, Empathy, or Intimacy. As such, each reaction was categorically coded, and could only be scored as one of the four themes. To examine the proportions of the main categories, scores for each category were summed across sessions. Each participant received a total score for each of the four major domains of level of personality functioning. A ratio (%) score for each domain was calculated based on each participant’s total amount of narrated reactions.

#### Symptomatology and Global Functioning at Baseline and End of Treatment

Personality problems, a broad spectrum of psychopathology, and global functioning were assessed at baseline and again at end of treatment (EOT).

The Borderline Personality Feature Scale for Children [32] is a 24-item self-report dimensional measure of borderline personality features for children aged 9 to 18 years. BPFS assesses four aspects of BPD (i.e., affective instability, identity problems, negative relationships, and self-harm) using six items, each rated on a five-point Likert scale (from 1 = “not at all true” to 5 = “always true”). All item scores are summed to yield a total score, with higher scores indicating greater levels of borderline features. The measure was administered in two versions, one for the adolescents (BPFS-C) and one for parents (BPFS-P) at baseline and end of treatment. The clinical cut-off score for BPFS-C is 66, and for BPFS-P 72 [33].

Participant’s externalizing and internalizing behavior were assessed using both parent’s and adolescent’s self-report. Parents completed the Child Behavior Checklist [34] consisting of 112 items scored on a three-point Likert scale (from 0 = “absent” to 2 = “occurs often”). Adolescents filled out the Youth Self-Report [35], which is the children’s version of CBCL, consisting of 112 items scored on a three-point Likert scale (from 0 = “not true” to 2 = “very true or often true”). The CBCL and YSR measure a broad range of different mental health disorders in young people aged 11 to 18 years. The internalizing dimension comprises the Anxious/Depressed, Withdrawn/Depressed, and Somatic Complaints scales. The Externalizing dimension comprises the Aggressive Behavior and Rule-breaking Behavior scales. Both CBCL and YSR have shown good psychometric properties [36].

Depression symptoms were measured using the Beck Depression Inventory for Youth [37] assessing negative thoughts, feelings of sadness, and physiological symptoms of depression. The BDI-Y consists of 20 items self-reported on a four-point Likert scale (from 0 = “never” to 3 = “always”) with higher scores indicating greater depressive symptoms. The BDI-Y was only administered to the adolescents. A Danish version of the BDI-Y was used, which has shown good internal consistency (α = 0.92 for girls) and high test–retest reliability (r > 0.70) [38].

Children’s Global Assessment Scale [39] assesses participants’ global level of functioning. The C-GAS score was based on information from the clinician-administered interview as well as medical records from the preceding month including data on hospitalizations, emergency room visits, and medication from patient’s medical accounts.

## 4. Results

### 4.1. Nature of Narrated Events: Agency and Communion

Adolescents would typically describe agency events about feeling down, exhausted, or a lack of control and achievement. Furthermore, agency events mostly took place in school settings in which the patients were anxious about attending school, absence from school, as well as change in everyday settings (e.g., moving home, moving school), see Table 1.

Communion events primarily encompassed romantic relationships (e.g., having a crush on someone, arguing with boyfriends, break ups), relationships with parents and family (e.g., parents being absent, arguments with parents), and friendships (e.g., lack of friends, supporting friends, and arguing with friends), see Table 1.

Across group sessions, unidentified events counted for 25%, given that patients were often allowed attending the sessions without sharing an event (see Table 2). There was a large variance across patients, with unidentified events counting from 12.5 to 41.2%.

**Figure 1:**
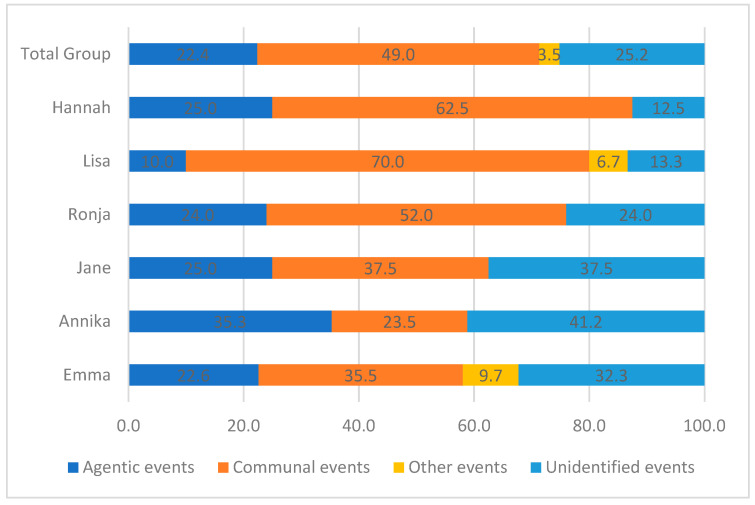
Ratio of themes in narrated events.

On an individual level, results showed that for five of six patients the main proportion of events encompassed communion themes, varying from 35.5 to 70.0%, with Hannah and Lisa displaying the highest proportions of communion-related events. The only exception was Annika, who in most sessions did not identify an event, and when telling an event, most often revolved around agentic themes (see Figure 1).

### 4.2. Change of Narrated Events: Agency and Communion

To explore changes related to narrative themes from the beginning to the end of therapy, we compared the adolescents first five sessions with their last five sessions using a Chi-Square Bhapkar Test of marginal homogeneity applicable for repeated nominal measures, see Table 2. The test showed a significant difference in the type of events. Compared to the first five sessions, the last five sessions of every participant showed an increase in agency events and in “other” events, a decrease in communion events, while the number of unidentified events (e.g., times in which the patients were not able to identify an event) stayed the same.

### 4.3. Nature of Narrated Reactions: Personality Functioning

**Figure 2:**
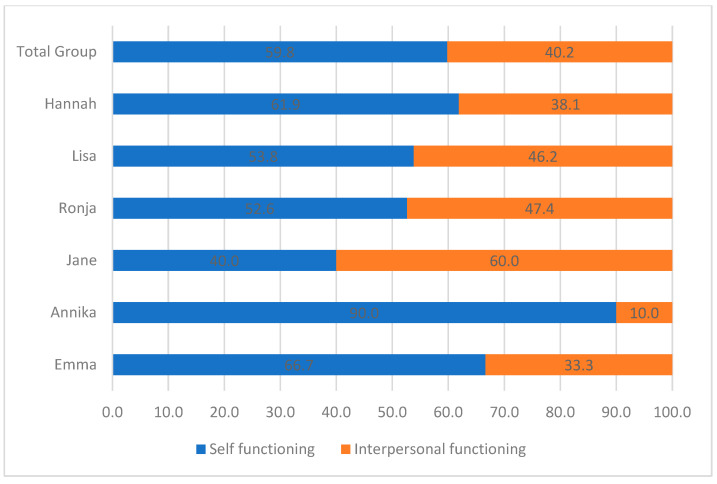
Ratio of personality functioning domains in narrated reactions.

**Figure 3:**
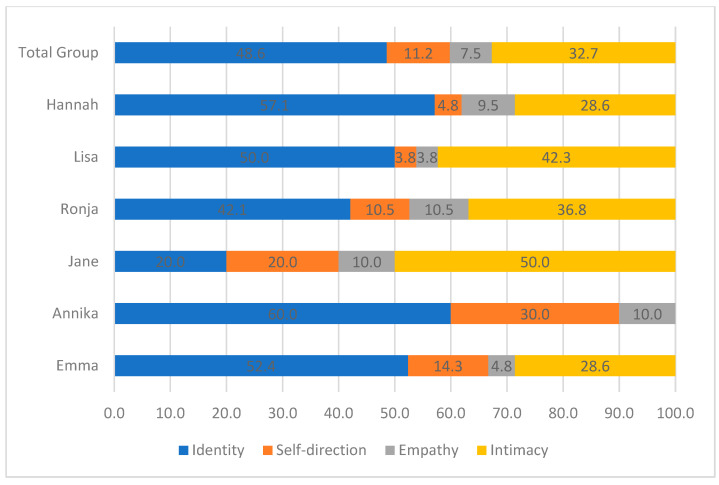
Ratio of personality functioning domains in narrated reactions.

The narrated reactions to the main events brought up in therapy were coded focusing on personality functioning (i.e., LPFS). As shown in Figure 2 and Figure 3, at the group level, narrated reactions primarily related to Self-Functioning and especially the domain of Identity. Reactions disclosing identity problems included severe emotion-regulation difficulties and breakdowns in self-coherence (see Table 3). On an individual level, five of the patients primarily narrated identity-related problems, varying from 42.1 to 60.0%. The only exception was Jane, who had an equal ratio of Identity and Self-direction struggles. That is, Self-Functioning and especially Identity were the key struggles in personality functioning expressed by patients as reactions to their narrated events.

Narrated reactions also disclosed struggles with Interpersonal Functioning, and primarily related to the domain of Intimacy. Specifically, patients expressed struggles with wanting closeness with others on the one hand, and on the other hand losing interest in or becoming scared of closeness (e.g., when feeling ignored by a partner). The adolescents predominantly showed the same ratio of narrated reactions disclosing Intimacy-related struggles. The only exception was Annika, who did not narrate any reactions related to Intimacy.

### 4.4. Change in Narrated Reactions: Personality Functioning

To compare the narrated reactions in the adolescents’ first five sessions with their last five sessions, a Chi-Square Bhapkar Test was conducted but showed a non-significant, difference. Thus, adolescents did not change the types of storied reactions to the events.

### 4.5. Symptomatology and Global Functioning

Differences in symptomatology and global functioning between baseline and end of treatment (EOT) were examined using a Paired Samples *t*-test (See Table 3).

There was a non-significant, moderate decrease in patients’ self-reported borderline features. However, the average end of treatment score still indicated clinically significant borderline features (BPFS-C > 66). There was a large near-significant drop in parent-reported borderline personality features (see Table 3).

For patients’ self-reports, there was a significant, large decrease in both externalizing and internalizing behavior, while change in depressive symptoms showed a non-significant, small effect. For parent reporting, there was a near-significant, large decrease in externalizing behavior and a significant, large decrease in internalizing behavior.

There was a significant, large improvement in clinician reports of the adolescents’ global functioning between baseline and end of treatment. However, the average C-GAS score of 47.7 at end of treatment indicated that the adolescents still showed a moderate degree of interference in functioning in most social areas, see Table 3.

## 5. Discussion

In this feasibility study, we were interested in focusing on the nature of, and change associated with, narrated events and the accompanying reactions from adolescents with BPD enrolled in an MBT-G treatment program. Themes of agency, communion, and “other” themes were identified. A total of 25% constituted unidentified events in which the adolescents were present but not able or willing to share an event, with high variance across patients (from 12.5 to 41.2%). Communion was the dominating theme for most participants (five out of six patients), but varied extensively from 35.5% to 70.0%. When comparing the participants’ first five sessions with their last five sessions, we found a significant increase in agency and in “other” themes and a decrease in communion themes related to the main events brought up in group therapy. The number of unidentified events did not change significantly.

With respect to the narrated reactions, most reactions centered around self-functioning and especially identity, but other-functioning in the form of intimacy was also prevalent. No significant shifts in reactions were observed when comparing the participants’ first five sessions with their last five sessions.

Finally, some significant changes in self-reported symptomatology and global functioning were found after MBT-G. The main findings will be discussed in the following section.

### 5.1. A Shift from Other-Functioning (Communion) towards a Focus on Self-Functioning in Both Nature and Reaction of the Narration

For some time, the field has been stressing the need to study narrative identity from new levels integrating novel timescales [40]. This study is a first attempt to observe narration within micro-interactions in group therapy in BPD. That is, while other studies examined changes in narrative identity from before to after therapy [29,41], this innovative study observed narrative shifts within the therapeutic session itself across group sessions. We identified a move from communion towards an increased focus on themes of agency—a finding that relates to extant literature showing increased agency in narrative identity in patients with BPD after completing psychotherapy [29,41]. Note that the current study did not focus on the valence of the themes but, instead, the dominance of specific narrative themes over others. Thus, the elevated presence of agency may not necessarily indicate improved agency, but certainly indicates an increased awareness of agentic themes in the patients’ lives. Other researchers have emphasized the importance of the self as a driver or nexus of personality pathology (e.g., [42]). The motivational narrative turn away from focusing on others and towards the self in the form of mastery, independence, and autonomy, even lack thereof, may be a necessary first step towards recovering. The adaptiveness of this turn might be reflected in the self-report measures, indicating that patients were less inclined to externalize their problems after therapy.

The phenomenological approach to assessing narrative identity themes has first and foremost provided us with invaluable insight into the type of daily struggles occupying adolescents with BPD. The normality of the tasks narrated is striking as they, to a large extend, reflect typical developing tasks in young individuals [27,43]. That is, the adolescents’ difficulties reflected in the narratives are prototypical to their developmental period for this age group (e.g., identity development, education, creating intimate bonds with others). Yet, their reactions to these developmental tasks (e.g., self-harm) deviate significantly from their non-BPD peers. Many individuals with BPD feel alienated from their surroundings [44] and it might therefore be worth normalizing the content of their struggles in therapy as they work with a therapist on more adaptive reactions to these challenges. Emphasizing links between the typical and atypical personality in the conceptualization and treatment of BPD are more relevant than ever as we embrace the new dimensional model to PD in ICD-11 [12]. The phenomenological approach also sheds light on the large heterogeneity of the patients reflected in the large variability within the narrative profiles. This may advocate for more individualized personal medicine in BPD, which seem to align well with the conceptualization of PD in ICD-11 [28].

### 5.2. Study Limitations

A number of study limitations should be acknowledged. The sample was small, and the feasibility study should therefore be replicated in a larger sample with a greater variety of PDs (not only BPD) and with an equal number of men and women. A larger sample will also enable the more advanced statistics necessary to examine relations between the coded variables and the self-report assessment. The sample comprised one of the more well-functioning groups of the larger study (i.e., fewer dropouts and fewer no-shows), which provided better conditions for coding. However, including a bigger sample will, and should, overcome this sample selection bias. Furthermore, empathy was less often identified than other themes of functioning. This is consistent with previous research [26,27], indicating that empathy is an aspect of personality functioning less often expressed explicitly in narratives and potentially more indirectly through intimacy. Finally, the newly developed coding system should be validated and compared to other well-established assessments on narrative themes [45] and personality functioning [46].

### 5.3. Concluding Remarks

In this feasibility study, we examined narrative identity as it is expressed within and across group sessions in adolescents manifesting BPD. We showed how some aspects of narrative identity stayed the same while others changed as therapy progressed, and how this phenomenological shift may also reflect the changes found in relation to self-reported results. The relevance of storying in group therapy was discussed and some implications flagged.

## Figures and Tables

**Table 1:** Type and number of narrative events, narrative reactions, and narrative exemplars.

Narrated Events		*n*	Examples
Agency themes			
	Generalized plots	15	“She felt that everyday life was boring” “She was in her room, and suddenly felt stressed out”
	School	10	“She had a panic attack at school, and does not want to go there anymore” “She had to meet with the headmaster due to truancy”
	Transitions	7	“She has been suggested to move to her cousins place in another city to start over” “She got told to move out of home”
Communion themes			
	Romantic relationships	33	“She had a fight with her boyfriend, because he texted with another girl” “She had started dating a new guy, but felt that he was moving too fast”
	Parents/family	24	“She visited her father” “She ended up discussing with her sister, because of guy”
	Friendships	13	“She tried to help a friend with relationship issues” “She went on a trip with a group of friends, but they ended up having a row”
Other themes		5	“She talked to her doctor about stopping medication” “She had her brace removed”
**Narrated Reactions**		***n***	**Examples**
Identity			
	Coherence	13	“She suddenly felt empty”
	Self-esteem	10	“His message made her feel weak”
	Emotion Regulation	26	“She ended up self-harming”
Self-direction			
	Goal-striving	12	“She could not decide whether she wanted to marry him”
	Internal standards of behavior	0	none
	Self-reflection	5	“She got confused about her feelings and did not know how to react”
Empathy			
	Comprehension of others’ experiences	4	“Her boyfriend did not answer her, because he was at school, which made her angry and anxious”
	Tolerance of others’ perspectives	2	“She felt she had wasted her time on him because he did not change opinion”
	Effect of one’s behavior on others	3	“She did not want to make her mom sad, so she stopped herself from self-harming”
Intimacy			
	Depth and duration	11	“She withdrew from him”
	Desire and capacity for closeness	14	“She turned to him to get calmed down”
	Reciprocity	7	“She scolded him, because she felt he was unrespectful”

**Table 2:** Comparison of narrated events and reactions from the five first and last sessions.

Events	*n* (%)	First 5 *n* (%)	Last 5 *n* (%)	χ^2^(3)	*p*
Total	143 (100)	30 (100)	30 (100)	8.8	0.03
Agency	32 (22.4)	5 (16.7)	11 (36.7)		
Communion	70 (49.0)	20 (66.7)	10 (33.3)		
Other	5 (3.5)	0 (0.0)	4 (13.3)		
Unidentified	36 (25.2)	5 (16.7)	5 (16.7)		
**Reactions**					
Total	107 (100)	30 (100)	30 (100)	5.7	0.13
Identity	52 (48.6)	15 (50)	13 (43.3)		
Self-Direction	12 (11.2)	1 (3.3)	7 (23.3)		
Empathy	8 (7.5)	3 (10.0)	1 (3.3)		
Intimacy	35 (32.7)	11 (36.7)	9 (30.0)		

**Table 3:** Clinical measures and comparisons between baseline and end of treatment.

Clinical Measures (*n* = 6)	Baseline M (SD)	EOT M (SD)	t(5)	*p*	Difference * (95% CI)	Hedges’ *g*
BPFS-C	86.2 (11.6)	76.8 (13.2)	1.69	0.15	9.3 (−4.9; 23.2)	0.58
BPFS-P	82.9 (14.2)	63.2 (15.4)	2.56	0.06	19.7 (−1.7; 41.1)	0.91
CBCL-P						
Externalizing	24.3 (12.7)	4.6 (9.2)	2.51	0.066	19.8 (−2.1; 41.7)	0.90
Internalizing	24.8 (10.8)	2.7 (6.0)	3.59	0.023	22.1 (5.0; 39.3)	1.3
YSR						
Externalizing	26.7 (7.6)	7.0 (4.3)	4.98	0.008	19.7 (8.7; 30.7)	1.8
Internalizing	34.6 (5.5)	12.8 (9.8)	4.26	0.013	21.8 (7.6; 36.0)	1.5
BDI-Y	30.9 (4.7)	29.0 (7.9)	0.51	0.634	1.9 (−7.9; 11.8)	0.17
C-GAS	37.5 (4.7)	47.7 (6.8)	−4.07	0.01	−10.2 (−16.6; −3.8)	−1.4

* = positive differences indicate an improvement. BPFS-C = Borderline Personality Feature Scale, children. BPFS-P = Borderline Personality Feature Scale, parents. CBCL-C = Child Behavior Checklist, self-report. CBCL-P = Child Behavior Checklist, parents. BDI-Y = Beck’s Depression Inventory for Youth. IPPA = Inventory of Parent and Peer Attachment. C-GAS = Children’s Global Assessment Scale. EOT = End of Treatment.

## Data Availability

Data is unavailable due to privacy restrictions.
